# Host competence of Algerian *Gerbillus amoenus* for *Leishmania major*

**DOI:** 10.1016/j.ijppaw.2023.04.007

**Published:** 2023-04-18

**Authors:** Kamal Eddine Benallal, Ghania Mezai, Mohammed Mefissel, Nadia Klari, Cylia Lardjane, Ahmed-Fayez Khardine, Ihcen Kherachi, Yacine Dib, Karima Brahmi, Jovana Sadlova, Petr Volf, Zoubir Harrat

**Affiliations:** a Laboratory of Parasitic Eco-Epidemiology and Genetic of Populations, Institut Pasteur of Algiers, Algeria; b Department of Parasitology, Faculty of Science, Charles University, Prague, Czech Republic; c Public Establishment of Nearby Health of Illizi, Ibn-Sina, Algeria; d Mouloud Maameri University, Department of Biology, Ecology and Biology of the Terrestrial Ecosystem Laboratory, Tizi Ouzou, Algeria; e Laboratory Emergent and Reemergent Viruses, Institut Pasteur of Algiers, Algeria

**Keywords:** *Leishmania major*, *Gerbillus amoenus*, Leishmaniasis, Rodents, Xenodiagnostic, qPCR, Algeria

## Abstract

Cutaneous leishmaniasis (CL) is the most important neglected disease reported in North Africa, Algeria ranks second in the world with more than 5000 cases per year. In Algeria, two rodent species *Psammomys obesus* and *Meriones shawi,* are so far known as proven reservoirs of *Leishmania major*, however, they are absent in several endemic localities. In this study, we experimentally infected *Gerbillus* rodents trapped around human dwellings in Illizi, Algeria to assess their susceptibility to *L. major*. Seven gerbils, morphologically and molecularly identified as *Gerbillus amoenus,* were intradermally inoculated with 10^4^ parasites derived from culture, monitored for six months and their infectiousness for sand flies was tested by xenodiagnosis. The study revealed that *G. amoenus* was susceptible to *L. major* and was able to maintain and transmit the parasites to sand flies tested six months after infection, suggesting the role of this gerbil as a potential reservoir for *L. major*.

## Highlights

• Occurrence of *Gerbillus amoenus* in Algeria.
• Susceptibility of *Gerbillus amoenus* to *Leishmania major* infections.
• Detection of the parasites' dissemination in rodent tissues using qPCR.
• Maintenance and transmission of parasites to sand flies after 6 months.

## Graphical abstract

**Figure:**
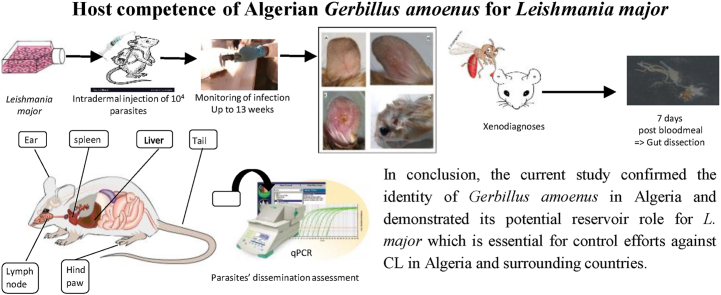

## 1 Introduction

Leishmaniases, regarded as neglected tropical diseases, are caused by protozoa belonging to the genus *Leishmania* (Kinetoplastida: Trypanosomatidae) (Valero and Uriarte, 2020). The disease represents a major health problem in many countries, with approximately 2 million new cases reported yearly (WHO, 2020). *Leishmania* is a parasite with a digenetic life cycle, alternating between blood-feeding insects, phlebotomine sand flies (Diptera: Psychodidae), and mammalian hosts including humans. Sand flies comprise more than 950 different species distributed in the Old and New World, they are incriminated in the transmission of various pathogens to humans, including *Leishmania* parasites, they also transmit the bacteria *Bartonella bacilliformis* and viruses belonging mainly to the genus *Phlebovirus* of the family Bunyaviridae (Benallal et al., 2022).

In the Maghreb region, CL is caused by three different *Leishmania* species: *L. major*, *L. tropica,* and *L. infantum* with distinct eco-epidemiology and clinical manifestation (Aoun and Bouratbine, 2014). The most frequent species is *L. major* transmitted by *Ph. Papatasi* and maintained mostly in two rodent species *Psammomys obesus* (Fat Sand Rat) and *Meriones shawi* (Shaw's Jird) (Belazzoug, 1986; Ghawar et al., 2011). However, in addition to these two proven reservoirs, other rodent species are likely to be involved in parasite maintenance, as evidenced by infections in areas where neither *M. shawi* nor *P. obesus* lives. Algeria has 27 other rodent species, some of which may play an important role in maintaining pathogens but they are not considered reservoirs of *Leishmania* parasites yet (Ahmim, 2019), moreover, the hedgehogs *Atelerix algirus* and *Paraechinus aethiopicus* were found naturally infected with *L. major* (Tomás-Pérez et al., 2014). Here we describe the possible involvement of another rodent species *Gerbillus amoenus* in the life cycle of *L. major*.

The genus *Gerbillus* is one of the most diverse groups of rodents occurring in arid and semi-arid areas (Abiadh et al., 2010). Recently, *Gerbillus nanus,* a cryptic species of the African *G. amoenus* (Ahmim, 2019; Ndiaye et al., 2016) was reported to be naturally infected by *L. major* in Iran (Azizi et al., 2011). In the central Sahara of Algeria, some sporadic outbreaks of CL have recently reemerged in the province of Illizi (INSP, 2017) where the proven reservoirs of *L. major* cited above do not occur (Ahmim, 2019) but rodents of the genus *Gerbillus* are abundant. Our study aimed to test the susceptibility of *Gerbillus* rodents to *Leishmania major* and to evaluate their role as reservoir hosts by assessing the maintenance and transmission of the parasites.

## 2 Material and methods

### 2.1 Study area

**Fig. 1:**
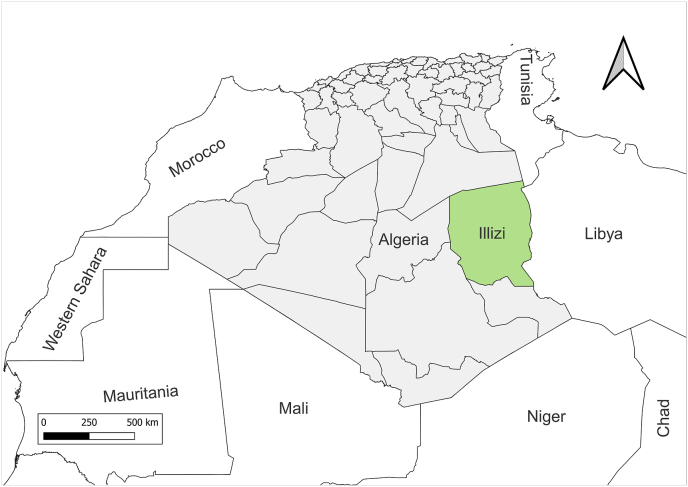
Location map of the study area.

The province of Illizi is located about 2200 km from the capital of Algeria (26°30′18″ N, 8°28′56″ E, 567 m above sea level) in the southern-eastern part of the central Sahara in Algeria and covers an area of 204.675 km^2^ (Fig. 1). The number of inhabitants is estimated to be 34.714 persons dispersed in 4 districts (Illizi, Debdeb, Bordj Omar Driss and In Amenas). Illizi is the gate to the Tassili N'Ajjer National Park and the environment is a mixture of rocky and sandy areas. Illizi has a hot desert climate with long, extremely hot summers and short cold winters (Beck et al., 2018). The average annual temperature is 33 °C. Illizi is virtually rainless throughout the year as the average annual rainfall is around 10 mm, the summers are especially dry and the relative humidity is very low with an annual average not exceeding 33%. The village of Indgidad-Imihrou; located about 180 km from the center of Illizi district; was chosen to conduct this study following the report in 2018 of two CL autochthonous cases. In this village, nomadic inhabitants live under huts made of stones and palm leaves.

### 2.2 Rodent trapping

In September 2019, 10 Sherman traps baited with fresh bread and roasted peanuts were set along dried rivers near active chicken coops, human inhabitants and animal shelters at sunset for three consecutive days. Traps were checked in the morning and the captured rodents were placed in cages for further analyses.

### 2.3 Breeding and molecular identification of the rodents

The trapped rodents were transferred to Algiers and handled at the Institut Pasteur of Algiers. As the institute's internal procedures recommended, all animals were left in quarantine for at least 2 months before entering the animal facility. Each animal was checked for the presence of ectoparasites (fleas and ticks) and the eventual presence of *Leishmania* lesions on the nose, ears, paws, and tail. Different body measurements (tail length, body length, hindfoot length, and ear height) were taken for morphological identification according to Granjon et al. and Ahmim (Ahmim, 2019; Granjon and Duplantier, 2009). One rodent was subjected to molecular identification targeting the mitochondrial gene Cyt B (Shenbrot et al., 2017).

The sequences were visualized and analyzed in BioEdit software (Hall, 1999) and then blasted against the GenBank database. The phylogenetic tree was drawn using the maximum likelihood method to estimate the evolutionary relationships among the sequences; General Time Reversible (GTR) and (G + I) was used as the best-fit model of nucleotide substitution according to the Bayesian Information Criterion (BIC) implemented in Mega 11 software (Tamura et al., 2021). The robustness of tree topologies in all the treatments was checked using 1000 bootstrap replicates and the Cyt B sequence of *M. shawi* was used as an outgroup. Pairwise Kimura 2-Parameters genetic distances were obtained for our Cyt B dataset under MEGA 11 (Tamura et al., 2021). The sequence of *Gerbillus amoenus* was submitted into the GenBank database under accession number OQ190728.

### 2.4 Parasite preparation

The virulent L. *major* strain MHOM/DZ/09/LIPA100/MON-25 isolated from a patient living in M'Sila province, Algeria was used. Promastigotes were cultured in a complete RPMI-1640 medium (Sigma-Aldrich) supplemented with 20% heat-inactivated fetal bovine serum (FBS) (Invitrogen Life Technologies, Villebon-Sur-Yvette, France). For experimental infection of the rodents, parasites in stationary phase culture were collected on day 6, washed three times in sterile saline, counted using a THOMA chamber, and re-suspended to a final density of 10^6^ promastigotes/ml.

### 2.5 Rodent infection and monitoring

Five female BALB/c mice (6 weeks old, weight between 18 ± 2 grs) and seven *Gerbillus* were selected for experiment infection. Rodents were anaesthetized with a mixture of ketamine/xylazine (66 mg/kg ketamine and 26 mg/kg xylazine) (“Ketamine/Xylazine,” 2006). 10 μL of the parasitic suspension corresponding to 10^4^ parasites were injected intradermally using a 27.5-gauge sterile needle with a diameter of 27.5 mm to the left ear pinna. The proportion of metacyclic promastigotes present in the inoculum was calculated using a Giemsa-stained smear of the suspension. Inflammation (red papule) appearing around the inoculation site was defined as swelling and a visible difference in the appearance of the infected ear, and skin crusting was defined as a lesion. The evolution of infection was monitored weekly in both rodent species by measuring the lesion diameter using a digital caliper (Schuster et al., 2014). Food and water were provided *ad libitum*, and the animals were maintained in standard conditions (12 h dark/12 h light photoperiod, at a temperature of 22–25 °C and humidity of 40–60%)**.**

### 2.6 Xenodiagnoses assay

Five to seven-day-old *Phlebotomus papatasi* sand fly females were used for xenodiagnosis experiments. The flies were maintained at 26 °C and 12/12 dark/light cycle as previously described (Volf and Volfova, 2011). The tests were performed six months post-infection only on *Gerbillus*, corresponding to the winter season in North Africa. Rodents were anaesthetized with a mixture of ketamine/xylazine (66 mg/kg ketamine and 26 mg/kg xylazine body weight, respectively), placed in small cages (20 × 20 × 20 cm) and exposed to 15–20 *Phlebotomus papatasi* females for 1 h. Engorged females were maintained at 26 °C on 50% aqueous sucrose solution (Volf and Volfova, 2011). On the 10th day post-bloodmeal (PBM) and after defecation, live females were dissected and their guts were examined under a light microscope. Intensities and locations of infections were evaluated as described previously (Sádlová and Volf, 2009). In dead females (engorged and gravid), *Leishmania* parasites were detected using PCR targeting kDNA and ITS-1sequences (Rodgers et al., 1990; Schönian et al., 2003).

### 2.7 Tissue sampling and quantitative PCR

Six months post-infection, the gerbils were sacrificed by injecting an overdose of ketamine/xylazine. Both ears (inoculated and contralateral), both ears-draining lymph nodes, spleen, liver, paws, and tail were stored at −20 °C for standard and quantitative PCRs. Extraction of total DNA from gerbil tissues was performed using a QIAamp DNA blood mini kit (QiaGen, Germany) according to the manufacturer's instructions. A first screen for *Leishmania* parasites in the tissue was carried out using conventional PCR targeting kDNA and ITS-1 sequences (Rodgers et al., 1990; Schönian et al., 2003) to select the positive samples for parasite quantification. In the second step, only the positive samples revealed by the conventional PCR were considered for qPCR quantification of *Leishmania* parasites load, the reactions were performed in Mic Real-Time PCR Cycler Systems using SuperScript III Platinium SYBR Green One-Step qRT-PCR Kit detection method (Invitrogen, Life Technology) using minicircle kinetoplast DNA as the molecular target (Myskova et al., 2008). As reported previously, the qPCR was considered specific when the melting temperature was 84 °C for the positive controls (Mihoubi et al., 2006).

### 2.8 Statistical analysis

The lesion diameters in the two groups of rodents were compared using a one-way ANOVA test using Microsoft Excel version 2013. The differences were considered significant if P < 0.05.

### 2.9 Animal experiment guidelines

Parasite Eco-epidemiology and Population Genetics laboratory at Institut Pasteur of Algiers has been authorized by the Algerian Association for Animals experimentation Science to carry out studies on rodent reservoirs of cutaneous leishmaniasis (Agreement N°45/DGLPAG/DVA/SDA/14).

## 3 Results

During the three nights of trapping, a total of 9 rodents (seven females and two males of *Gerbillus*) were captured, the trap efficiency corresponds to 0.3 rodents/trap/night. Unfortunately, only females survived the trip to the laboratory located about 2000 km from Illizi and were not pregnant. The morphological identification of nine *Gerbillus amoenus* (2 males and 7 females) according to Ahmim (2019) (Suppl. information 1) was confirmed by sequencing of the Cyt B gene. Indeed, the maximum likelihood (ML) tree showed that the gerbil belonged to *G. amoenus* as it clustered within the monophyletic group of *G. amoenus* from Mali, Morocco, Mauritania, Egypt and Niger (Suppl. information 3) showing genetic distance ranging between 0.002 and 0.015, and differed from *G. nanus* which showed genetic distances ranging from 0.066 to 0.071 (Suppl. information 2).

Visual screening carried out on the nose, ears, paws, tail did not reveal presence of *Leishmania* lesions suggesting the absence of parasitic infection. In addition, no ectoparasites were found in the trapped rodents.

### 3.1 Infection lesion monitoring

**Fig. 2:**
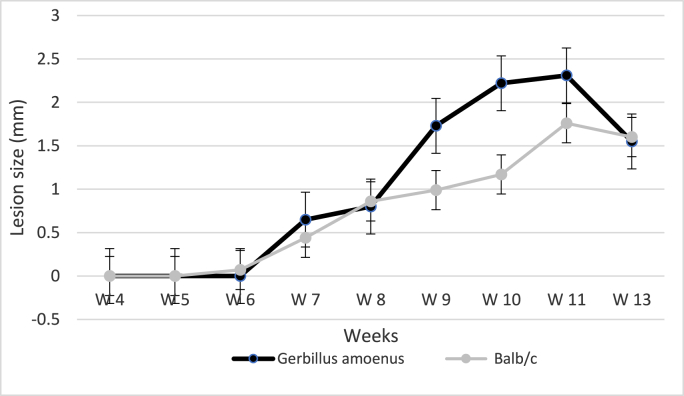
**Lesion growth in *Gerbillus amoenus* and Balb/c mice.** Data are presented as the means ± standard errors of the means.

The inoculum contained 25% metacyclic promastigote forms meaning that the infections were triggered by 2500 metacyclic promastigotes. At the fourth week post-infection, the rodents showed swelling on the inoculation sites which evolved to lesions from week 6 and week 7 in BALB/c mice and gerbils, respectively (Fig. 2). The lesion diameters increased to reach a peak of 2.31 mm in gerbils and 1.76 mm in mice at week 11. In both groups, monitoring was stopped at week 13 since it was impossible to measure the lesion diameters in the ears that started to necrotize. Comparison of lesion size between the two groups of rodents showed no statistically significant difference (P = 0.51, F = 0.4595) suggesting that in this respect, both groups of rodents have the same susceptibility to the *L. major* parasites.

### 3.2 Xenodiagnoses

**Fig. 3:**
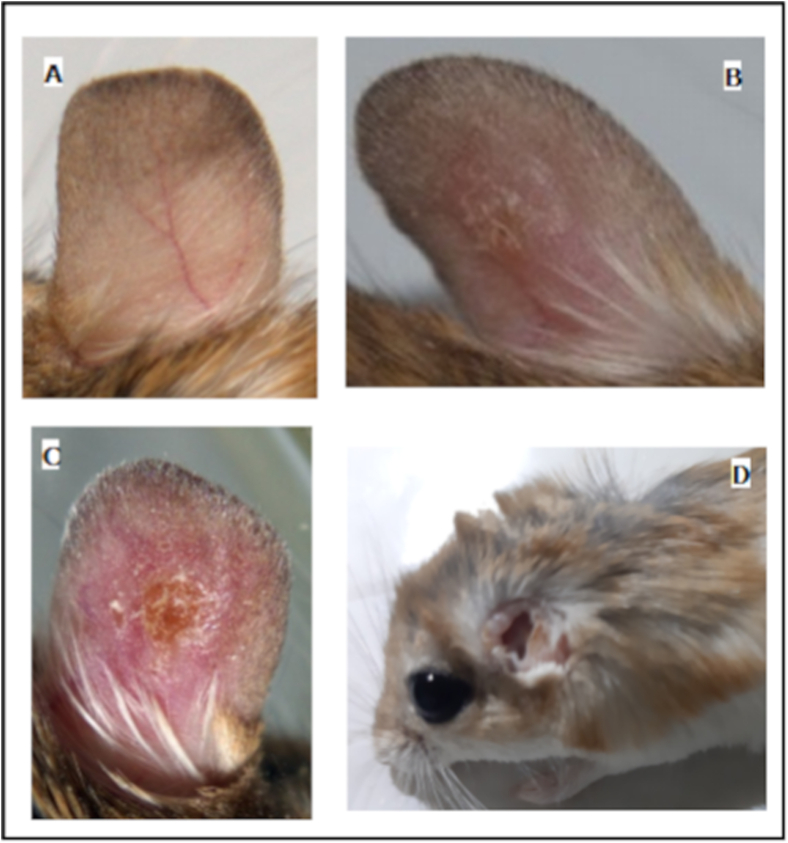
The external manifestation of *L. major* infection in *Gerbillus amoenus.* A) non-infected ear, B) 8th-week post-infection, C) 11th-week post-infection, D) 6 months post-infection.

Three gerbils died before the end of the experiment, only four individuals served to the xenodiagnoses test. Six months post infection, the infected ears of surviving gerbils were already damaged by parasites (Fig. 3D). A total of 77 female sand flies were left to feed on four anaesthetized rodents (Each rodent was exposed separately to 22–25 sand fly females). Ten days PBM, only 10 female sand flies survived, one was positive for *Leishmania* parasites and showed a moderate parasite density in the thoracic midgut and the 67 dead females were tested by PCR, two of which were confirmed positive for *Leishmania* parasites. In total, 3.89% of 77 *Phlebotomus papatasi* females tested positive and infected on three different rodents.

### 3.3 Distribution of L. *major* in rodent bodies

In qPCR, the Ct of the negative samples was 31, all samples with a Ct ≥ 30 were considered negative. QPCR detected the parasites in all tested organs and tissues, but mostly in low numbers. Therefore, the positivity of the samples was double-checked using two PCR techniques (conventional and quantitative PCR) thus giving accuracy and insurance to our results. An exception was in gerbil N°4 which presented a high parasite load in the spleen, tail, and hind paws (Table 1).

**Table 1:** qPCR detection of *Leishmania* parasites in gerbil tissues.

Rodent	Left inoculated ear (LE)	Right ear (RE)	LE draining LN	RE draining LN	Liver	Spleen	Tail	Hind paws
1	NA	<10	261	NA	12	33	107	98
2	NA	<10	34	NA	20	11	30	51
3	11	<10	NA	<10	<10	<10	105	38
4	NA	<10	84	<10	<10	2037	2751	7454

NA: not analyzed (left ears were mostly destroyed by infection), LN: lymph node.

## 4 Discussion

The genus *Gerbillus* is one of the most diverse rodent groups occurring in arid and semi-arid areas (Abiadh et al., 2010; Dahmana et al., 2020). *Gerbillus amoenus* (Blanford, 1875) has been reported from Morocco to Egypt, while its cryptic species *G. nanus* is widespread from the Middle East and the Arabian Peninsula to Asia (Ndiaye et al., 2016). So far, the rodent populations of Algeria have not been genetically studied in detail. Indeed, molecular identification of the gerbil in this study confirmed the occurrence of *G. amoenus* as the analyzed specimen was placed within the monophyletic group of *G. amoenus* from countries bordering Algeria such as Morocco, Libya, Mauritania, Mali and Niger as shown in Supplementary information 2. Thus, the molecular result confirms the deep molecular and karyotype analyses previously performed by Ndiaye (Ndiaye et al., 2016) and completes the range of this species in North Africa.

This study, to our knowledge, is the first to assess the susceptibility and reservoir role of *G. amoenus* to *L. major*. The experimental infections performed showed that *G. amoenus* are susceptible to *L. major*, similar to BALB/c mice. In both species (BALB/c mouse and *G. amoenus*), parasites caused ear lesions and spread to different body parts including viscera. The spread of *Leishmania* throughout the gerbils’ body has been described in other experimental infections of potential African reservoir hosts of *L. major* – rodents of the genera *Mastomys* and *Arvicanthis,* however, in these sub-Saharan species, *L. major* did not cause skin lesions and destruction of the pinnae, the parasites produced only slight skin changes such as hyperpigmentation (Sadlova et al., 2020). Importantly, *G. amoenus* maintained the parasites and were infectious to sand flies even after six months suggesting that this species meets the most important criteria of a reservoir host previously described (Chaves et al., 2007). *Gerbillus amoenus* is known to share burrows with the proven reservoir species of *L. major* such as *Meriones libycus* and *Psammomys obesus* (Ahmim, 2019; Tabbabi, 2019) which increases the risk of its natural infections with *Leishmania* parasites. In addition, this species can be used as a laboratory animal model for *L. major* infections. Similar to the Asian rodent species *Phodopus sungorus*, *Lagurus lagurus* and *Cricetulus griseus*, these wild rodents are genetically polymorphic and better mimic human infections than inbred mice strains (Vojtkova et al., 2020).

In conclusion, the current study confirmed the identity of *Gerbillus amoenus* in Algeria and demonstrated its potential reservoir role for *L. major* which is essential for control efforts against CL in Algeria and surrounding countries.

## Funding

This study was funded by the internal research funding of the Institut Pasteur of Algiers LeisHeild-Mati project N° 04–2018 and 10.13039/501100001824Czech Science Foundation (GAČR, project N° 23-06299S). Supply and shipment of sand flies from Prague to Algiers was funded by The European Union's Horizon 2020 Research and Innovation Program under grant agreement No 731060 (Infravec2).

## Author's contributions

Kamal Eddine Benallal: designed, trapped, infected, analyzed data, wrote the manuscript and acquired funding, Ghania Mezai: cultivated parasites, Mohamed Meffisel and Yacine Dib: helped in rodent trapping, Karima Brahmi: helped in data analyses, Nadia Klari and Cylia Lardjane: rodent monitoring and data analyses, Ahmed-Faize Khardine: sequencing and qPCR analysis, Ihcen Kherachi: helped in experimental infection of rodents, Jovana Sadlova: reviewed and edited the manuscript, Petr Volf and Zoubir Harrat: reviewed, edited the manuscript and acquired funding.

## Declaration of competing interest

The authors declare that they have no known competing financial interests or personal relationships that could have appeared to influence the work reported in this paper.
