# Supplementary material for: Host competence of Algerian Gerbillus amoenus for Leishmania major
Source: Int J Parasitol Parasites Wildl. 2023 Apr 18;21:69–73. doi: 10.1016/j.ijppaw.2023.04.007 (PMC10151220; doi:10.1016/j.ijppaw.2023.04.007)
Supplement: Multimedia component 1 [file mmc1.docx]

**Suppl. Table 1**: Body measurements (in mm) of the trapped rodents:

|  | Head + body | Tail | Hindfoot | Ear | Weight (gr) | Sex |
| --- | --- | --- | --- | --- | --- | --- |
| *Gerbillus amoenus* N°1 | 85 | 105 | 25 | 11 | 25 | Female |
| *Gerbillus amoenus* N°2 | 85 | 112 | 23 | 12 | 18 | Female |
| *Gerbillus amoenus* N°3 | 85 | 111 | 22 | 11 | 19 | Female |
| *Gerbillus amoenus* N°4 | 100 | 110 | 22 | 13 | 22 | Female |
| *Gerbillus amoenus* N°5 | 87.5 | 107 | 21.5 | 11.5 | 24 | Female |
| *Gerbillus amoenus* N°6 | 89 | 106 | 22.6 | 12.3 | 23.5 | Female |
| *Gerbillus amoenus* N°7 | 91 | 108 | 23.2 | 11.2 | 24.5 | Female |
| *Gerbillus amoenus* N°8 | 78 | 101 | 21 | 10.2 | 17 | Male |
| *Gerbillus amoenus* N°9 | 80 | 103 | 21.3 | 10.6 | 18.5 | Male |
