# Supplementary material for: Host competence of Algerian Gerbillus amoenus for Leishmania major
Source: Int J Parasitol Parasites Wildl. 2023 Apr 18;21:69–73. doi: 10.1016/j.ijppaw.2023.04.007 (PMC10151220; doi:10.1016/j.ijppaw.2023.04.007)
Supplement: Multimedia component 2 [file mmc2.docx]

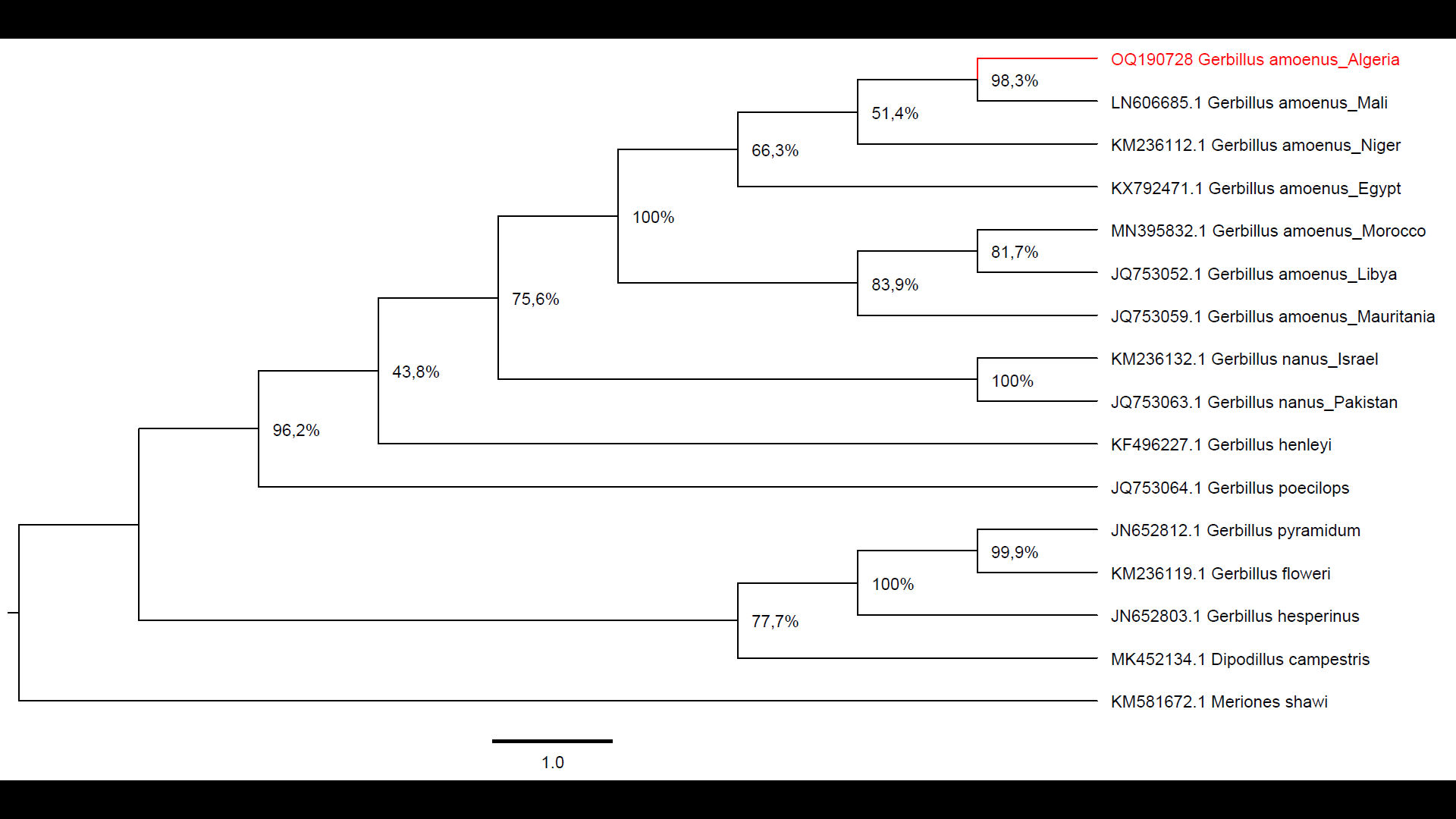


Suppl. Information 2: Phylogenetic reconstruction based on 1100 bp sequences of the cytochrome b gene using the maximum likelihood method. Values on nodes correspond to bootstrap values. The coloured taxon corresponds to the Algerian specimen sequence and the rest was downloaded from NCBI database based on (Bouarakia et al., 2019).

Bouarakia, O., Denys, C., Nicolas, V., Benazzou, T., & Benhoussa, A. (2019). First molecular identification of *Gerbillus amoenus* (Rodentia, Muridae) in Morocco. *Zoology and Ecology*, *29*(2), 106–112. https://doi.org/10.35513/21658005.2019.2.6
